# Rasch Analysis of a New Hierarchical Scoring System for Evaluating Hand Function on the Motor Assessment Scale for Stroke

**DOI:** 10.1155/2014/730298

**Published:** 2014-08-07

**Authors:** Joyce S. Sabari, Michelle Woodbury, Craig A. Velozo

**Affiliations:** ^1^College of Health Related Professions, Occupational Therapy Program, State University of New York, Downstate Medical Center, 450 Clarkson Avenue, Brooklyn, NY 11203, USA; ^2^Ralph H Johnson VA Medical Center, Charleston, SC 29425, USA; ^3^Department of Health Sciences and Research, Medical University of SC, Charleston, SC 29425, USA; ^4^Division of Occupational Therapy, Medical University of South Carolina, Charleston, SC 29425, USA

## Abstract

*Objectives*. (1) To develop two independent measurement scales for use as items assessing hand movements and hand activities within the Motor Assessment Scale (MAS), an existing instrument used for clinical assessment of motor performance in stroke survivors; (2) To examine the psychometric properties of these new measurement scales. *Design*. Scale development, followed by a multicenter observational study. *Setting*. Inpatient and outpatient occupational therapy programs in eight hospital and rehabilitation facilities in the United States and Canada. *Participants*. Patients (*N* = 332) receiving stroke rehabilitation following left (52%) or right (48%) cerebrovascular accident; mean age 64.2 years (sd 15); median 1 month since stroke onset. *Intervention*. Not applicable. *Main Outcome Measures*. Data were tested for unidimensionality and reliability, and behavioral criteria were ordered according to difficulty level with Rasch analysis. *Results*. The new scales assessing hand movements and hand activities met Rasch expectations of unidimensionality and reliability. *Conclusion*. Following a multistep process of test development, analysis, and refinement, we have redesigned the two scales that comprise the hand function items on the MAS. The hand movement scale contains an empirically validated 10-behavior hierarchy and the hand activities item contains an empirically validated 8-behavior hierarchy.

## 1. Introduction

To maximize functional outcomes, occupational therapists and physical therapists assess and provide interventions related to gross mobility, sitting and standing balance, ambulation, and motor performance of the arm and hand. Rehabilitation clinicians use standardized assessment tools on a daily basis to determine patients' baseline performance, guide treatment planning, monitor ongoing progress, establish recommendations for follow-up care after discharge, and evaluate the effectiveness of interventions.

The Motor Assessment Scale (MAS) [1] provides rehabilitation clinicians and researchers with a single, quickly administered assessment of eight categories of poststroke motor function: supine-to-sidelying; supine-to-sitting; balanced sitting; sitting-to-standing; walking; upper arm function; hand movements; and advanced hand activities. Each category is scored on a 7-point scale (0–6), based on a person's ability to perform specific tasks. The tasks in each category are intended to be hierarchical; that is, the ability to accomplish task 6 implies the ability to accomplish tasks 1 through 5. This arrangement reduces administration time and increases its appeal to clinicians.

The MAS is a well-respected instrument [2], with excellent test-retest reliability [1], interrater reliability [1, 3], concurrent validity with both the Fugl-Meyer Assessment [3–5] and the Action Research Arm Test [4, 6]. Because of its format, therapists report that it is efficient to use and helpful in planning interventions [7]. It has been used as a primary outcome measure in studies of standard neurorehabilitation approaches [8], electrical stimulation [9, 10], virtual reality [11], circuit training [12], constraint-therapy [13], and type of rehabilitation facility [14, 15]. Additionally, it has been used in a variety of descriptive studies related to stroke rehabilitation [16–18].

As a clinical evaluation tool, the MAS is preferable to other assessments that are often selected for use in stroke research studies. The Wolf Motor Function Test [19] and Arm Motor Ability Test [20] require extensive setup and are prohibitively lengthy for routine clinical use. The Action Research Arm Test [21] requires purchase of a costly test kit. Self-report measures, such as the Stroke Impact Scale [22] and Motor Activity Log [23] require accurate reflection by patients about daily activity patterns and may be most appropriate for individuals who have already completed the early stages of stroke rehabilitation. The Fugl-Meyer Assessment [24], generally accepted as the gold standard in stroke research, has excellent reliability and validity, but it is lengthy to administer, it can be criticized for being based on outdated theoretical constructs [25, 26], and it measures movement impairments exclusively, with no evaluation of functional performance [3].

From a measurement perspective, the eight items on the MAS may be viewed as eight separate scales, each scored according to a person's ability to pass a series of six dichotomously scored behavioral criteria. The behavioral criteria are arranged in hierarchical difficulty order, in which a score of 0 indicates inability to achieve any of the criteria and a score of 6 indicates ability to perform all the criteria. When administering the MAS, a therapist assigns a score for each item, based upon the most difficult criterion the patient can achieve. Total MAS scores represent the sum of scores for each of the 8 items and range from 0 to 48. Studies report the total score for all 8 items [8, 14], total score for only the mobility, balance and walking items [15], or scores for single items, such as walking [16, 18, 27] or upper arm [8, 9]. Often, the score for the upper limb (UL) subscale [11–13, 17, 28, 29] is reported. This score, which can range from 0 to 18, is based on performance in the upper arm, hand movements, and advanced hand activities items.

The MAS' unique organization is efficient because it enables therapists to assign scores for each item without assessing all six behavioral criteria. The assumption is that if a patient can achieve the criterion for a score of 6 (or 5, etc.), the person is also able to achieve the criteria for all lower scores. Therefore, this structure requires a valid difficulty-level hierarchy for the behavioral criteria subsumed within each of the 8 items on the MAS.

The scoring criteria for the bed mobility, balance, sit-to-stand, and walking items are characterized by clear performance hierarchies. For example, the hierarchical score for sitting-to-standing is determined by the amount of assistance needed, symmetry of weight distribution, and performance speed. Similarly, the hierarchical score for balanced sitting is based on graded increments of displacements of the person's center of mass. Since the hierarchical criteria for scoring the upper limb items are less obvious, several researchers have questioned [3, 5, 30–33] the behavioral hierarchies for items 6 (upper limb), 7 (hand movements), and 8 (advanced aand activities).

We were the first to use Rasch analysis to assess the hierarchal arrangement of behaviors comprising the UL subscale items [30]. In a 100-person sample of stroke patients, we found that each of the 3 items was unidimensional. However, only the upper arm item's behavioral criteria could be empirically supported (i.e., the item-difficulty hierarchy proposed by the original authors was supported by the Rasch measurement model). The original hierarchy of behaviors for the hand movements and advanced hand activities items was not empirically supported by the Rasch measurement model. Two subsequent studies supported the scoring criteria for the upper arm but not advanced hand activities [32, 33] and one of these also concurred with our finding about hand movements [33].

In summary, the MAS, overall, is a useful and efficient tool that is preferable to other assessments for evaluating a range of motor behaviors in stroke survivors. However, problems in the scoring criteria for the hand items are a negative factor that decreases the tool's appeal to clinicians and researchers. In our previous publication [30], we recommended improving the two hand items by (1) clearly differentiating between movements and activities; (2) adding criteria that would be appropriate for stroke survivors with lower levels of hand function; and (3) providing a valid difficulty hierarchy for behavioral criteria. The purpose of this study was to develop two independent measurement scales, hand movements and hand activities, for use in clinical practice and outcomes research. Here, we report on the multistep process of developing and examining the psychometric qualities of these new items.

## 2. Materials and Methods

### 2.1. Preliminary Scale Development

We used both methodological and clinical perspectives in determining a pool of behavioral criteria associated with hand movements and with hand activities, with the goal of developing a psychometrically valid instrument that can also provide clinicians with guidelines for treatment. Several early decisions were based upon our previous analysis of dimensionality and hierarchies of difficulty for the original behavioral criteria on items 7 and 8 of the MAS [30].

The first decision was to clearly delineate between hand movements and hand activities. To most occupational therapists and physical therapists, “movement” refers to a person's capacity to actively produce a change in position of a specific body part, and “activity” refers to a person's capacity to integrate movements for performance of functional tasks. In describing hand dysfunction in stroke survivors, Raghavan [34] distinguishes between deficits in* motor execution*, associated with specific paralysis, and deficits in* motor control*, associated with motor planning and motor learning. When assessing and intervening to improve hand function after stroke, rehabilitation therapists are interested in knowing which active movements an individual can produce and which types of grasp a person is able to use for functional task performance. The original hand movement item on the MAS assesses forearm, wrist, and thumb motions and includes two behavioral criteria (picking up a large ball and picking up a polystyrene cup) that we would consider to be* activities*, rather than* movements*. On making this clearer distinction between items 7 and 8 on the MAS, we adopted the name hand activities (instead of advanced hand activities) to clearly differentiate this item from item 7, hand movements, and to remove the implication that all behaviors related to hand activities will be more difficult, as compared to all behaviors related to hand movements.

The second decision was to eliminate significant influences of proximal arm function on a person's ability to achieve a passing score on the items assessing hand function. This decision led to removing those behavioral criteria which required the individual to combine hand performance with antigravity shoulder and elbow movements, such as reaching forward, moving a utensil to one's mouth, and combing hair at the back of one's head.

From a theoretical perspective, we were interested in testing two commonly held beliefs: (1) that a particular movement would be easier when performed in a gravity-eliminated plane and more difficult when performed in an antigravity plane; and (2) that certain types of grasp are easier to achieve than others. Therefore, on the criteria for our revised hand movements scale, we included adaptations to standard assessments that are typically used in manual muscle testing (with two items for each movement—one against gravity and the other in a gravity-eliminated plane) [35]. On the criteria for our revised hand activities scale, in addition to tasks from the original MAS involving holding and using a writing implement, we included tasks that inherently require a person to perform lateral pinch [36] (pick up a penny by sliding it to the edge of the table), pad to pad pinch [36] (pick up a pencil), tip to tip pinch [36] (pick up a penny from the table), and cylindrical grasp [36] (grasp a 0.5 liter bottle). Finally, we made sure to include only items which any individual, without hand impairments, could easily achieve with either the dominant or nondominant hand.

The next step in preliminary development of behavioral criteria was to examine our previous findings related to floor and ceiling effects, as well as large gaps in difficulty levels between sequential behaviors tested on the advanced hand activities item. In particular, we had identified a need to provide behaviors that would be easier to achieve than picking up and putting down a pen cap. We selected a behavioral task involving activation of a remote control device, as well as a task in which the person would be required to use the hand for stability purposes only (moving paper on a table).

Finally, some changes to the original criteria on the advanced hand activities item were based on practical rationales. We changed picking up the top of a pen and putting it down again to picking up and putting down a standard lead pencil since pencils have more consistent size and shape, as compared to the tops of pens, and a pencil is used in subsequent writing tasks on the assessment. We also removed the item involving balancing water on a spoon since water may not be readily available and spills could create the need for clean-up. An item requiring that the person pick up a jellybean from a cup was eliminated because jellybeans are impractical to keep in a test kit over lengthy periods of time.

Our preliminary scale development resulted in 14 criteria assessing hand movements and 10 criteria assessing hand activities. We deliberately included more than the required 6 criteria for each category, with the goal that Rasch analysis would reveal the 6 criteria that provide the best hierarchy of difficulty levels.

### 2.2. Initial Pilot Testing to Refine the Research Instrument

We conducted two phases of initial pilot testing for the purpose of maximizing interrater reliability (IRR). In the first phase of pilot IRR study, 15 occupational therapists at an urban hospital participated. Training consisted of watching a video showing the administration of the assessment to healthy individuals. Afterwards, the occupational therapists watched and independently scored a video showing the administration of the assessment to three stroke survivors at different stages of recovery. In addition, they provided narrative feedback about the clarity of test directions and scoring criteria for each behavioral item. We calculated percentage of exact agreement (PEA) with gold standard scores established by the researchers. PEA for individual items varied between 66.7% and 100%. Average PEA for the hand movements scale was 93%. Average PEA for the hand activities scale was 94%. Using both narrative and PEA data, we improved the test protocol and scoring directions and produced two new videos, consisting of vignettes from a total of 8 post-stroke individuals performing the test items. In the second phase of pilot IRR study, 82 occupational therapists from four urban hospitals participated. Training consisted of watching a video showing selected vignettes of testing poststroke survivors and distribution of the test protocol for therapists to practice. Two weeks later, therapists watched a second video of different selected vignettes and independently scored a patient's performance on each item. Narrative comments improved significantly, and PEA scores rose to an average PEA of 95.7% for hand movements and 96.3% for hand activities. PEA for individual items varied between 84.1% and 98.8%.

### 2.3. Data Collectors and Training

Thirty occupational therapists at eight clinical sites in the United States and Canada participated in preliminary training before serving as data collectors.

The PI assigned one member of each site's team to serve as site coordinator, and the PI provided written test protocols and complete test kits (see Section 3) to each site coordinator. In addition, the PI provided each site coordinator with training and examination videos.

Prospective data collectors participated in video-based training in which they learned the test protocol and had the opportunity to view vignettes of patient performance to illustrate examples of behaviors that would constitute passing and failing each item. Following this, prospective data collectors completed a rater qualification exam based on their ratings of a videotaped, volunteer stroke survivor performing each item. Before beginning data collection, raters were required to achieve a minimum of 90% agreement with gold standard scores. An additional videotape of a second volunteer stroke survivor's performance was available in the event any data collector needed to have a second opportunity to pass the rater qualification exam. The mean score on rater qualification exams was 98.78% in agreement with gold standard scores.

### 2.4. Materials

In addition to the training materials, The PI provided test kits to each participating site. Each test kit consisted of a printed test protocol, score sheets for data collection, and the following items:

- blank 8(1/2) × 11′′ paper (1 sheet/assessment);
- 8(1/2) × 11′′ paper with vertical lines, drawn 1′′ away from each vertical side (2 sheets/assessment);
- 0.5 liter empty water bottle;
- 8 oz. disposable cup (3.5′′ height);
- standard number 2 pencil;
- US penny;
- stop watch;
- universal remote control device.

### 2.5. Procedure

IRB approvals were secured at eight hospital and rehabilitation facilities in the United States and Canada, and the study was conducted in accordance with each site's approved procedures. Subjects were recruited from the general population of stroke rehabilitation inpatients and outpatients at each facility. Individuals were included in the study if they exhibited some ability to move or use their paretic hand. All testing took place in the occupational therapy treatment areas within the participating facilities. Before testing, the data collector recorded each participant's age, gender, hand tested (left or right), whether the tested hand was previously dominant or nondominant for writing tasks, and time since the individual had sustained the stroke.

Seated at a standard table, each participant used the paretic hand to perform the 14 behavioral criteria comprising the hand movements scale and then performed the 10 behavioral criteria comprising the hand activities scale. The data collector recorded “Pass” or “Fail” for each behavior, based on standardized scoring criteria in the study protocol. Completed score sheets were sent to the PI for data entry.

### 2.6. Data Analysis

We used the dichotomous Rasch model (Winsteps software) [37] to assess dimensionality, test reliability, and hierarchy of difficulty for behavioral criteria on the hand movements and hand activities scales.

#### 2.6.1. Dimensionality

The extent to which the criteria contributed to a unidimensional construct was examined with goodness-of-fit statistics and principal components analysis of the residuals.

*(1) Goodness-of-Fit Statistics*. Under the Rasch model, goodness-of-fit statistics evaluate the assumption that low-ability persons should do well on easy criteria and poorly on difficult criteria, while higher-ability persons should do well on both easy and difficult criteria. We examined the infit statistic which is sensitive to performance of behaviors closely matching subjects' ability. Fit statistics are reported as a mean square of the standardized residuals (MnSq) with an associated* z*-value (ZStd). Acceptable psychometric criteria for unidimensionality were defined as MnSq values 0.5–1.7 and ZStd < 2.0 [30].

*(2) Principal Components Analysis of the Rasch-Generated Residuals*. According to the Rasch model, all information contained in the dataset should be explained by the underlying construct intended (i.e., hand movements or hand activities) If true, then the unexplained aspects of the data, the residuals, cannot be explained by another trait and are simply random noise. Unidimensionality is supported when the variance explained by the first factor >50%, the unexplained variance <5.0%, and the eigenvalue for the first residual factor <2.0 [38].

#### 2.6.2. Reliability

One purpose of patient evaluation is to describe how one patient's skills are different from another patient's skills. Acceptable Rasch person-separation index values are >2.0 [39]. Person strata indicate the number of “centers” that are three measurement errors apart. A minimum of two strata would assure that the hand movements and activity scales were reliably sensitive enough to distinguish between people with low versus high ability in the sample.

#### 2.6.3. Hierarchy of Behavioral Criteria

Through Rasch measurement, items represent “difficulty” markings (calibrated in log equivalent units or logits) along a metric representing the continuum of the underlying construct. By placing person-ability scores on this same metric, Rasch analysis directly links person-ability and item-difficulty [39]. When applied to the hand movements and hand activities scales, the analysis arranged the behavioral criteria according to difficulty so that the easiest criteria were those that were performed by individuals with “poor” hand skills and the most difficult were those that were performed by individuals with “good” hand skills.

#### 2.6.4. Item Refinement

The final step in translating our work to the development of improved items for the MAS was to reduce the number of behavioral criteria on each scale. Thus, we selected six behaviors per scale that represented a range of difficultly levels that matched the range of the sample's ability level. We avoided items that were of similar item-difficulty levels. We reintroduced behaviors one-by-one while reapplying Rasch analysis at each step until the person-separation value ≥2.0 and dimensionality criteria were adequate. We then determined the precision of the finalized scales by calculating the number of significant strata identified in the sample.

## 3. Results

### 3.1. Participants

Three hundred thirty-two stroke survivors participated. Participants ranged in age from 18 to 97 years (s.d. = 15 years) with a mean and median of 64 years. Of those whose gender was reported, 153 (48%) were female; 168 (52%) were male. Of those whose affected side was reported, 162 (52%) had left hemiparesis; 146 (48%) had right hemiparesis. Of those whose dominance was reported, 132 (44%) were tested with their dominant hand; 168 (56%) were tested with their nondominant hand. Time since stroke ranged from 1 day to 26 years. Although this seems like a very wide range, the median time since stroke was one month. One hundred ninety-four participants (68%) had sustained the stroke <= one month prior to testing; 285 participants (86%) had sustained the stroke within one year prior to testing. Severity of motor impairment ranged from individuals who could only perform minimal hand movement to individuals who had recovered significant function in the paretic hand.

### 3.2. Dimensionality

#### 3.2.1. Goodness-of-Fit

Behavioral criterion fit statistics for the hand movements and hand activities items are presented in Tables 1 and 2. All criteria exhibited acceptable infit values.

#### 3.2.2. Principal Components Analysis

For the hand movements item, the first dimension explained 71.30% variance, the unexplained variance was 4.6%, and the eigenvalue of first residual factor was 2.3. for the hand activities item, the first dimension explained 80.90% variance, the unexplained variance was 2.6%, and the eigenvalue of first residual factor was 1.4. While the eigenvalue for the hand movements item is above criterion of 2.0, since the explained variance is over 15 times greater than the unexplained variance, we considered the item as essentially unidimensional.

### 3.3. Reliability

The person-separation values for the hand movements and hand activities items were 2.17 and 2.16, respectively, which indicates that each of the items separates the sample into three ability strata.

### 3.4. Hierarchy of Behavioral Criteria

Criteria in the hand movements and hand activities items are arranged hierarchically in Tables 1 and 2. The difficulty calibrations and standard errors (logits) are shown in the rightmost column of each table. The hierarchical arrangement of each item's behavioral criteria can also be visualized on the item-person maps presented in Tables 3 and 4. When we examined the sample's performance of the easiest criteria, we found that, on the hand movements item, 96% of the sample achieved the easiest criterion, finger flexion with gravity eliminated, and 92.5% achieved the second easiest criterion, any thumb motion. For the hand activities item, 94.3% achieved the easiest criterion, slide paper, and 78.9% achieved the second easiest criterion, use remote control.

### 3.5. Item Refinement

#### 3.5.1. Hand Movements Item

We selected six behavioral criteria from the hand movements item that ranged in difficulty; (1) mass finger flexion with gravity eliminated, (2) any thumb motion, (3) wrist extension against gravity, (4) wrist radial deviation against gravity, (5) timed thumb opposition, and (6) finger intrinsic movement against gravity. Rasch analysis of these six criteria revealed an unacceptable person-separation value of 1.28. Therefore, movement behaviors were reintroduced one-by-one and the person-separation was recalculated until a value of 2.0 was obtained. This resulted in a set of 10 behavioral criteria shown in Table 5 which exhibited adequate measurement properties: person-separation of 2.11, acceptable infit values, strata of 3.15, and acceptable principal components analysis results. The first dimension explained 71.92% variance, unexplained variance was 4.2%, and the eigenvalue of first residual factor was 1.7. The sample's scores ranged from 0.0 to 10.0 with a mean of 7.0 and standard deviation of 2.6.

#### 3.5.2. Hand Activities Item

As in the procedure described above, we chose six behavioral criteria from the hand activities item that ranged in difficulty; (1) slide paper, (2) use remote control, (3) pick up pencil, (4) place penny in a cup, (5) timed dots, and (6) timed horizontal lines. Rasch analysis of these 6 behaviors revealed an unacceptable person-separation value of 1.52. Therefore, behavioral criteria were reintroduced one-by-one and the person-separation was recalculated until a value of 2.0 was obtained. This resulted in a set of 8 behavioral criteria shown in Table 6 which exhibited adequate measurement properties: person-separation of 2.31, person strata of 3.41, acceptable infit values, and acceptable principal components analysis results. The first dimension explained 82.30% variance, unexplained variance was 3.1%, and the eigenvalue of first residual factor was 1.4. The sample's scores ranged from 0.0 to 8.0 with a mean of 4.7 and standard deviation of 2.4.

## 4. Discussion

We present two amended items for assessing hand function on the MAS: hand movements, consisting of 10 behavioral criteria, and hand activities, consisting of 8 behavioral criteria. In addition to providing a clear differentiation between motor execution and motor control [26], we have added easier behavioral criteria which will enable the items to be used with patients demonstrating more severe impairment. These criteria are as follows: slide paper, use remote control, and use lateral pinch to pick up a penny. To prevent development of learned nonuse [40, 41], it is important for rehabilitation therapists to identify early capacities in patients' ability to move and use the paretic hand in order to develop creative ways for stroke survivors to use their paretic limb during daily activities. Most importantly, each item's behavioral criteria are not only unidimensional and reliable but also arranged in an empirically derived hierarchal difficulty order. This is important for the MAS because item scores depend on the validity of hierarchical arrangement of the behavioral criteria used to score the item. The behavioral criteria defined in this study allow a therapist to be reasonably confident that if a patient can achieve the criterion for a particular score on a selected item, the therapist does not need to test the criteria for lower scores.

### 4.1. Hand Movements Item

The hierarchical arrangement of behavioral criteria for the hand movements item is consistent with the assumption that movements are easier when performed in a gravity-eliminated plane and more difficult when performed in an antigravity plane. Mass finger flexion, wrist extension, wrist radial deviation, and the lumbrical/interossei action of simultaneously flexing at the metacarpophalangeal (MCP) joints while maintaining full extension at the proximal interphalangeal (PIP) joints were all more difficult to achieve when performing against gravity. This finding supports the conventional wisdom that, when designing therapeutic tasks to improve hand function in stroke survivors, it is wise for therapists to first introduce tasks that require the patient to use emerging movements in gravity-eliminated planes.

When developing the behavioral criteria for this item, we had hypothesized that the lumbrical/interossei action would be less difficult for stroke survivors than timed thumb opposition. Thus, we expected that these criteria would be useful in filling in the gap we found in difficulty level between timed thumb opposition (the most difficult item, scored as “6” on the initial version of the MAS) and the next most difficult item for our sample (radial deviation). Our finding that antigravity performance of IP extension combined with MP flexion in the four digits was more difficult for the sample group we tested was surprising. Although there is no literature to support this, we propose two conjectures that may explain this finding. The first is that this is a particularly novel movement that is unfamiliar to most individuals, whereas thumb opposition to all the digits is a movement sequence that many stroke patients may intuitively practice as they recover motor innervation to the intrinsic thenar muscles. A second possible reason why this particular movement may be more difficult than timed thumb opposition is that, in order to flex the MCP joints while simultaneously extending the IP joints of digits 2–4, the individual is faced with a dual motor challenge—to recruit the intrinsic lumbrical and interossei muscles while also inhibiting contraction of the extrinsic finger flexors.

### 4.2. Hand Activities Item

The hierarchical arrangement of behavioral criteria in the hand activities item provides information regarding the difficulties of various grasp patterns. Overall, we found that movements created by extrinsic hand muscles are easier than movements requiring intrinsic muscle function. Specifically, using the thumb to depress a key on a remote control device can be performed through use of Flexor Pollicis Longus—which is consistent with reports from data collectors that “any thumb motion” (on the movements item) was, most typically, flexion at the first MCP and PIP joints. In the sample of stroke survivors we tested, lateral pinch was the next easiest grasp pattern, followed, in order of successive difficulty, by cylindrical grasp, pad to pad pinch, tip to tip pinch, and skilled control of a pencil for untimed, and finally timed writing tasks.

### 4.3. Scoring the Items

To conform to the 0–6-point ordinal scoring system used by all other items on the MAS, we propose the following scoring system for the hand movements and hand activities items (see Tables 7 and 8). As in other items on the original MAS, individuals will score 6 points for successfully performing the most difficult motor behavior and 3 points if they are able to perform the behavior associated with the midpoint of motor abilities assessed by the item. Other scores are prorated to provide appropriate increments for achievement of increasingly more demanding behaviors on the 10-level hand movements item and the 8-level hand activities item. This scoring system permits clinicians and researchers to comply with the MAS' intended score range of 0–48 points [2] and/or the score range of 0–18 points for the upper limb subscale [20].

### 4.4. Study Limitations and Recommendations

Although the sample reflects a normal distribution of demographic variables, there is no way of knowing whether it reflects a representative sample of motor abilities after stroke. Since the final scale compositions were determined by removing and adding items with the criteria of achieving unidimensionality and a person separation of 2.0, the finalized scales need to be tested on an independent sample to determine whether these psychometric criteria are maintained.

For the hand movements item, future researchers might also consider testing potential behavioral criteria that would allow for more precise assessment of people functioning at higher levels than an ability to perform wrist radial deviation, but not quite as proficient as being able to perform the finger intrinsic motion of MCP flexion combined with IP extension. Possible criteria might include timed sequences of finger tapping.

## 5. Conclusions

The MAS uses a hierarchical scoring system that enables rehabilitation therapists to efficiently assess a range of motor abilities required for stroke survivors to function in daily activities. The behavioral hierarchies for bed mobility, balance, sit-to-stand, and walking items are based on a logical progression of abilities. The behavioral hierarchies for the assessment of upper arm function have been tested and validated [30, 32, 33]. However, the behavioral hierarchies for scoring the hand items have been criticized for inaccuracies in the postulated sequence of difficulty [3, 5, 30–33].

Following a multistep process of scale development, refinement, and Rasch analysis, we have redesigned the two hand items on the MAS. Our proposed hand movements and hand activities items measure a unidimensional construct and can reliably measure individuals with different levels of ability. In addition, the hierarchical ordering of behaviors proposed to score each item is supported by Rasch analysis.

## Figures and Tables

**Table 1:** Behavioral criteria statistics for hand movements item.

	Dimensionality				Item difficulty
	Infit MnSq	ZStd	Outfit MnSq	ZStd	Measure (standard error)
Finger intrinsic against gravity	0.77	−2.50	9.90	9.90	6.53 (0.21)
Timed thumb opposition	1.43	4.10	1.58	1.50	5.90 (0.19)
Finger intrinsic gravity elim.	0.61	−4.60	9.90	9.90	5.30 (0.19)
Wrist radial dev. against gravity	1.23	1.70	1.94	2.00	1.83 (0.21)
Wrist radial dev. gravity elim.	1.09	0.60	0.49	−1.10	0.04 (0.24)
Wrist extension against gravity	0.79	−1.50	0.37	−1.50	−0.83 (0.25)
Finger extension gravity elim.	0.78	−1.60	0.24	−2.10	−1.02 (0.26)
Finger extension against gravity	0.81	−1.30	0.25	−2.10	−1.05 (0.26)
Wrist extension gravity elim.	0.74	−2.00	0.27	−2.00	−1.22 (0.26)
Forearm supination	0.99	0.00	0.31	−1.80	−1.50 (0.26)
Any thumb motion	1.16	1.00	0.68	−0.50	−2.80 (0.30)
Forearm pronation	1.11	0.60	0.35	−2.00	−3.60 (0.34)
Finger flexion against gravity	0.91	−0.40	9.90	9.90	−3.60 (0.34)
Finger flexion gravity elim.	1.01	0.10	0.38	−1.80	−3.98 (0.37)

**Table 2:** Behavioral criteria statistics for hand activities item.

	Dimensionality				Item difficulty
	Infit MnSq	ZStd	Outfit MnSq	ZStd	Measure (standard error)
Timed horizontal line	1.02	0.2	0.88	−0.20	9.20 (0.34)
Timed dots	0.70	−1.8	0.50	−1.80	5.74 (0.25)
Horizontal line	0.76	−1.4	1.79	1.30	1.41 (0.26)
Make a mark	0.61	−2.8	0.45	−1.40	0.54 (0.26)
Penny in cup	1.18	1.1	4.31	4.30	0.54 (0.26)
Pick up pencil	0.70	−2.5	0.25	−2.50	−1.04 (0.24)
Pick up penny (slide to edge)	0.82	−1.6	0.35	−2.30	−2.74 (0.22)
Pick up bottle	0.89	−0.9	0.45	−2.00	−2.94 (0.22)
Use remote control	0.99	−0.1	8.20	9.70	−3.71 (0.23)
Slide paper	1.37	1.4	9.90	9.90	−6.99 (0.37)

**Table 3:** Person-behavioral criteria map for hand movements.

Hand movements		
Higher ability people	Logits	More difficult behavioral criteria
∗##########	7	
∗########		**Finger intrinsic against gravity**
∗	6	
		**Timed thumb opposition**
∗		
		**Finger intrinsic gravity eliminated**
∗#######		
	5	
∗	4	
∗		
∗###########		
	3	
	2	
		**Wrist radial dev.against gravity**
∗###		
	1	
∗##		
	0	**Wrist radial dev.gravity elim.**
∗#		
#		**Wrist extension against gravity**
	−1	Finger extension gravity elim.
		**Finger extension against gravity**
		Wrist extension gravity elim.
∗#		**Forearm supination**
∗		
∗#	−2	
∗#		
		**Any thumb motion**
	−3	
∗#		
		Forearm pronation
		Finger flexion against gravity
∗		
	−4	**Finger flexion gravity eliminated**
∗		
∗	−5	

Lower ability people	Logits	Less difficult behavioral criteria

In this map of person abilities and behavioral criteria, each # indicates 4 people and each ∗ indicates 1–3 people. These representations of person abilities are on the left and behavioral criteria are on the right. Lower ability people and easier behavioral criteria are at the bottom of the map and higher ability people and more difficult behavioral criteria are at the top of the map.

**Table 4:** Person-behavioral criteria map for handa.

Hand Activities		
Higher ability people	Logits	More difficult behavioral criteria
#######	10	
		**Timed horizontal line**
	9	
	8	
∗############		
	7	
	6	
		**Timed dots**
	5	
#############	4	
	3	
	2	
∗###		
		**Horizontal line**
	1	
		Make a mark
		**Penny in cup**
∗##		
	0	
∗###		
	−1	**Pick up pencil**
#####		
	−2	
∗####		
		**Pick up penny (slide to edge)**
	−3	Pick up bottle
		**Use remote control**
∗######	−4	
	−5	
∗		
∗######		
	−6	
∗#	−7	**Slide paper**

Lower ability people	Logits	Less difficult behavioral criteria

In this map of person abilities and behavioral criteria, each # indicates 4 people and each ∗ indicates 1–3 people. These representations of person abilities are on the left and behavioral criteria are on the right. Lower ability people and easier behavioral criteria are at the bottom of the map and higher ability people and more difficult behavioral criteria are at the top of the map.

**Table 5:** Behavioral criteria statistics for 10-behavior short form of hand movements item.

	Dimensionality				Item difficulty
	Infit MnSq	ZStd	Outfit MnSq	ZStd	Measure (standard error)
Finger intrinsic against gravity	0.78	−2.40	9.90	9.90	5.57 (0.21)
Timed thumb opposition	1.45	4.20	1.50	1.20	4.93 (0.19)
Finger intrinsic gravity elim.	0.62	−4.50	9.90	9.90	4.32 (0.19)
Wrist radial dev. against gravity	1.18	1.30	1.90	1.90	0.82 (0.21)
Wrist radial dev. gravity elim.	0.92	−0.50	0.44	−1.30	−0.92 (0.24)
Wrist extension against gravity	0.76	−1.80	0.36	−1.50	−1.75 (0.25)
Finger extension against gravity	0.91	−0.60	0.37	−1.50	−1.96 (0.25)
Forearm supination	0.85	−1.0	0.28	−1.90	−2.40 (0.26)
Any thumb motion	1.04	0.30	0.56	−0.80	−3.69 (0.30)
Finger flexion gravity elim.	0.99	0.00	0.61	−0.90	−4.91 (0.39)

**Table 6:** Behavioral criteria statistics for short form of hand activities item.

	Dimensionality				Item difficulty
	Infit MnSq	ZStd	Outfit MnSq	ZStd	Measure (standard error)
Timed horizontal line	1.02	0.20	0.91	−0.10	8.75 (0.34)
Timed dots	0.68	−2.00	0.32	−2.30	5.28 (0.25)
Horizontal line	0.78	−1.40	1.67	1.20	1.13 (0.25)
Place penny in cup	1.00	0.00	3.50	3.40	0.33 (0.25)
Pick up pencil	0.63	−3.10	0.23	−2.80	−1.19 (0.24)
Pick up penny (slide to edge)	0.72	−2.50	0.26	−2.50	−2.94 (0.23)
Use remote control	0.96	−0.30	6.84	8.50	−3.97 (0.24)
Slide paper	1.27	1.00	9.90	9.90	−7.39 (0.39)

**Table 7:** Scoring the hand movements item.

Score	Behavioral criterion
6.0	Finger intrinsic against gravity
5.4	Timed thumb opposition
4.8	Finger intrinsic gravity eliminated
4.2	Wrist radial deviation against gravity
3.6	Wrist radial deviation gravity eliminated
3.0	Wrist extension against gravity
2.4	Finger extension against gravity
1.8	Forearm supination
1.2	Any thumb motion
0.6	Finger flexion gravity eliminated
0.0	Unable to perform any motion

**Table 8:** Scoring the hand activities item.

Score	Behavioral criterion
6.0	Timed horizontal lines
5.25	Timed dots
4.50	Horizontal line
3.75	Place penny in cup
3.00	Pick up pencil
2.25	Pick up penny (slide to edge)
1.50	Use remote control
0.75	Slide paper
0.00	Unable to perform any activity

## List of Abbreviations

MAS: Motor assessment scale
UL: Upper limb
PEA: Percent of exact agreement
MnSq: Mean square standardized residual: observed variance ÷ expected variance
MCP: Metacarpophalangeal
PIP: Proximal interphalangeal
Grav Elim: Gravity eliminated
Rad Dev: Radial deviation.
